# Clinical-radiomics-based treatment decision support for KIT Exon 11 deletion in gastrointestinal stromal tumors: a multi-institutional retrospective study

**DOI:** 10.3389/fonc.2023.1193010

**Published:** 2023-08-14

**Authors:** Yu Zhang, Xiaofei Yue, Peng Zhang, Yuying Zhang, Linxia Wu, Nan Diao, Guina Ma, Yuting Lu, Ling Ma, Kaixiong Tao, Qian Li, Ping Han

**Affiliations:** ^1^ Department of Radiology, Union Hospital, Tongji Medical College, Huazhong University of Science and Technology, Wuhan, China; ^2^ Hubei Province Key Laboratory of Molecular Imaging, Wuhan, China; ^3^ Department of Gastrointestinal Surgery, Union Hospital, Tongji Medical College, Huazhong University of Science and Technology, Wuhan, China; ^4^ Department of Radiology, The First Affiliated Hospital of Guangxi Medical University, Nanning, China; ^5^ He Kang Corporate Management (SH) Co. Ltd., Shanghai, China

**Keywords:** radiomics, CT, gastrointestinal stromal tumor, KIT exon 11, nomogram

## Abstract

**Objective:**

gastrointestinal stromal tumors (GISTs) with KIT exon 11 deletions have more malignant clinical outcomes. A radiomics model was constructed for the preoperative prediction of KIT exon 11 deletion in GISTs.

**Methods:**

Overall, 126 patients with GISTs who underwent preoperative enhanced CT were included. GISTs were manually segmented using ITK-SNAP in the arterial phase (AP) and portal venous phase (PVP) images of enhanced CT. Features were extracted using Anaconda (version 4.2.0) with PyRadiomics. Radiomics models were constructed by LASSO. The clinical-radiomics model (combined model) was constructed by combining the clinical model with the best diagnostic effective radiomics model. ROC curves were used to compare the diagnostic effectiveness of radiomics model, clinical model, and combined model. Diagnostic effectiveness among radiomics model, clinical model and combine model were analyzed in external cohort (n=57). Statistics were carried out using R 3.6.1.

**Results:**

The Radscore showed favorable diagnostic efficacy. Among all radiomics models, the AP-PVP radiomics model exhibited excellent performance in the training cohort, with an AUC of 0.787 (95% CI: 0.687-0.866), which was verified in the test cohort (AUC=0.775, 95% CI: 0.608-0.895). Clinical features were also analyzed. Among the radiomics, clinical and combined models, the combined model showed favorable diagnostic efficacy in the training (AUC=0.863) and test cohorts (AUC=0.851). The combined model yielded the largest AUC of 0.829 (95% CI, 0.621–0.950) for the external validation of the combined model. GIST patients could be divided into high or low risk subgroups of recurrence and mortality by the Radscore.

**Conclusion:**

The radiomics models based on enhanced CT for predicting KIT exon 11 deletion mutations have good diagnostic performance.

## 1 Introduction

Gastrointestinal stromal tumor (GISTs) is the primary stromal tumor of the gastrointestinal tract (1). Most GISTs were found to contain mutations that constitutively activate the proto-oncogene receptor tyrosine kinase (RTK) KIT (2). Because activating alterations in KIT impair the natural autoinhibitory status of RTKs (3), leading to aberrant RTK, GISTs have become a model for successful molecular targeted treatment (4). Several small molecular compounds that target the KIT protein, such as imatinib (5), sunitinib (6), and regorafenib (7), are effective in treating advanced GISTs and have been approved for the treatment of advanced GISTs. All KIT inhibitors are widely used for patients with advanced GISTs and significantly improve the survival of patients (8, 9). Therefore, it is crucial to make an accurate diagnosis of GISTs so that optimal treatment can be used for patients with GISTs.

As the response to treatment varies substantially depending on the location of the mutation in GIST patients, the gene mutations status of the tumor is extremely important (10). GISTs with KIT exon 11 deletions have more malignant clinical outcomes (11, 12), and these deletions have a negative prognostic impact on recurrence-free survival (13–15). Predicting the progression of GIST is a vital aspect of providing good counseling and treatment to patients. Particularly, accurate prognostication is essential for identifying tumors with significant risk that need appropriate adjuvant systemic treatment. Recent research (16) on patients who had surgery for a localized GIST indicated that the chance of recurrence was exaggerated more than 30% of the time. In contrast to patients who benefited from a correct estimate of recurrence risk, these people got insufficient treatment and had a much greater incidence of relapse (17, 18). The presence of a KIT exon 11 deletion might be an additional factor for more precise patient identification for adjuvant treatment.

Samples from preoperative fine-needle aspiration biopsies are used in the traditional method for measuring KIT exon 11 mutation before surgery or any other treatment. However, the KIT exon 11 mutation evaluation depending on invasive biopsy could not accurately reflect all GISTs and has limited use in the preoperative evaluation of GISTs due to the small size and normal gastric mucosa covering of samples (19). In addition, biopsies of gastric GISTs may cause tumor rupture and dissemination (20) Thus, gastric GISTs are often evaluated by radiology (21, 22).

Radiomics is a non-invasive method for predicting the status of gene by radiomics, particularly for tumor heterogeneity (23). Previous research has demonstrated that radiomics has a high degree of precision in evaluating the entire biological activity of GISTs, particularly their potential for malignancy (24–26) and recurrence (27). In this research, a clinical-radiomics nomogram that intuitively describes the relationship between the variables in the prediction model was established and validated to predict the KIT exon 11 mutation status of GIST patients.

## 2 Materials and methods

### 2.1 Participants

**Figure 1:**
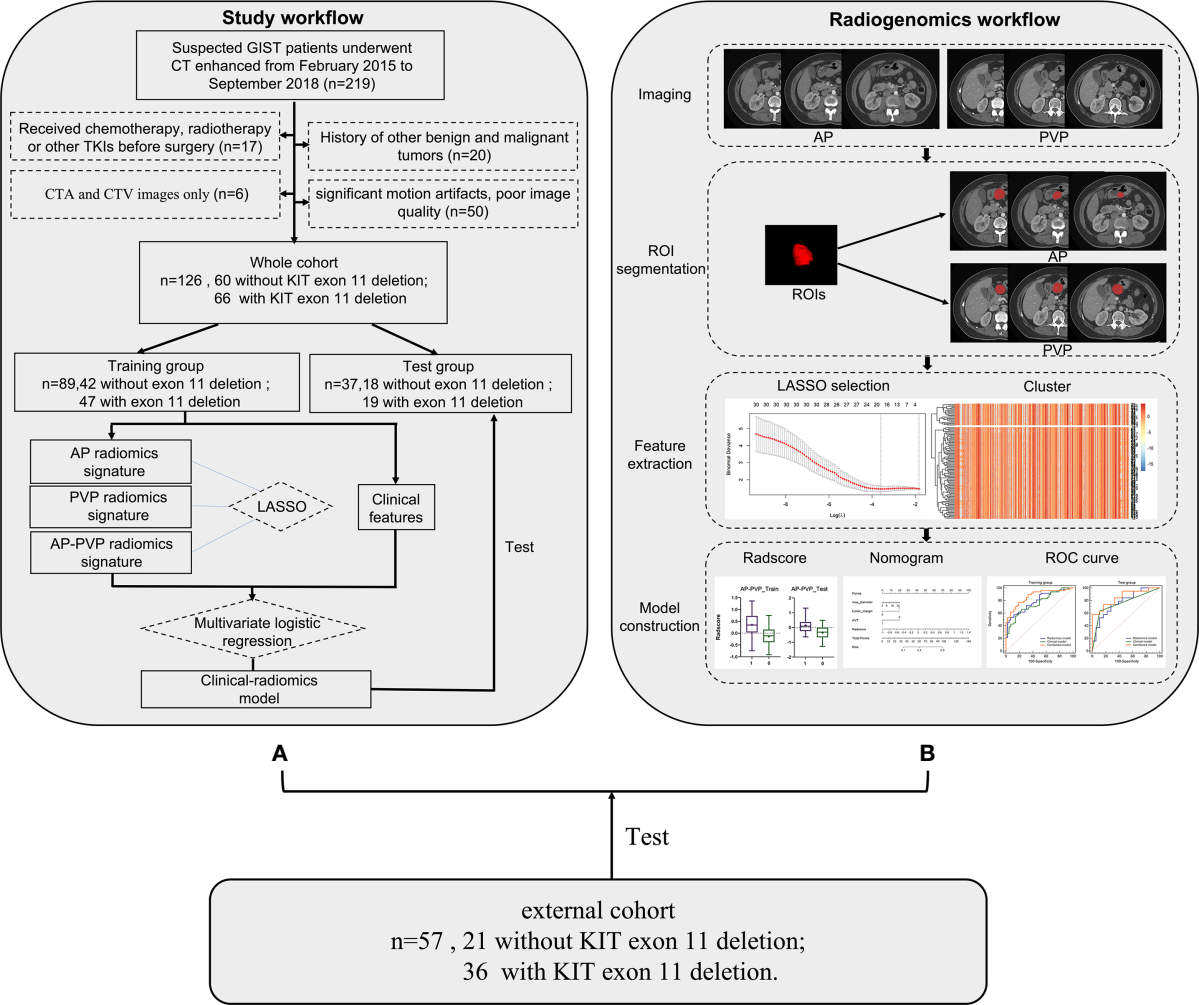
Workflow process for radiomics processing and analysis in this study. **(A)** Study flowchart. **(B)** Radiomics workflow. GIST, gastrointestinal stromal tumor; CT, computed tomography; ROI, region of interest; LASSO, least absolute shrinkage and selection operator.

A total of 126 patients (75 Males with mean age 53.8 years) with GISTs from February 2015 to September 2018 were retrospectively enrolled to predict the KIT exon 11 mutation status of GIST patients. The inclusion and exclusion criteria were seen in **Table 1**. The patient screening process is shown in the study flowchart in **Figure 1A**. This study is a retrospective study based on data from one of our clinical studies, and ethical approval was obtained by the Ethics Committee of Tongji Medical College of Huazhong University of Science and Technology. Written informed consent was obtained from all patients.

**Table 1:** Inclusion and exclusion criteria of patients in the training and test cohort.

Inclusion criteria	Exclusion criteria
localized primary GIST patients who underwent surgical resection with curative intent.	patients received chemotherapy, radiotherapy, imatinib therapy or other TKIs before surgery.
GISTs confirmed by postoperative pathology and immunohistochemistry examinations.	history of other benign and malignant tumors.
contrast-enhanced computed tomography (CECT) performed within 15 days before the surgery.	CTA and CTV images only.
complete clinical and pathological data available.	significant motion artifacts, poor image quality, or gastrointestinal mesenchymal lesions that could not be accurately evaluated.

57 patients with GISTs were retrospectively enrolled as an external cohort with the same inclusion criteria. The external cohort patients were enrolled from The First Affiliated Hospital of Guangxi Medical University.

### 2.2 Image acquisition

All subjects fasted for more than 4 hours before the computed tomography (CT) examination (Scanners: Aquilion ONE, Toshiba Medical Systems, Tokyo, Japan; SOMATOM Definition AS+/Definition, Siemens Healthineers, Erlangen, Germany; Discovery CT750 HD, GE Healthcare, Milwaukee, WI; IQon, Philips Healthcare, Best, the Netherlands). After a non-contrast CT scan with a thickness of 1.25 mm to 2.0 mm was performed, a dynamic contrast-enhanced scan was performed with 90-120 ml of iodine contrast medium (Visipaque, 320 mgI/mL, GE Healthcare Ireland, Shanghai) injected intravenously at a flow rate of 2.0 to 3.0 ml/s. The arterial phase and portal venous phase images were obtained with a delay of 25–30 s and 50–70 s after the injection. The parameters of CT scanning were as follows: tube voltage 100–120 kV; automatic tube current; slice thickness 1.25-2.0 mm; and standard algorithm.

### 2.3 Genetic testing

All patients were identified by genetic testing for the KIT exon 11 mutation. and the details of genetic testing are provided in **Supplementary Text S1**.

### 2.4 Image preprocessing and image quantization

All these images should be pre-processed before radiomics features extracted. All images have been normalized by MATLAB (RRID: SCR_001622). The details of the image preprocessing and image quantization are provided in **Supplementary Text S2**.

### 2.5 Region of interest segmentation and feature extraction

The radiomics workflow is shown in **Figure 1B**. The tumors were selected as the regions of interest (ROIs), which were segmented on arterial phase and venous phase images through the open-source software ITK-SNAP layer by layer (version 3.6.1, www.itksnap.org). The ROIs were delineated manually by 3 radiologists. The segmentations of X.F.Y. with 4 years of diagnostic experience and L.X.W. with 3 years of diagnostic experience were compared for interobserver differences. All images were segmented by another radiologist with 10 years of experience (Y.Z.) who was blinded to the type of GIST, repeated measurements were performed at an interval of 2 weeks, and the segmentations were compared for intraobserver differences. The intraobserver and interobserver differences were assessed by calculating the intraclass correlation coefficient (ICC) and features with consistency values<0.7 were removed. Finally, radiomics features were extracted using the ROI of the first segmentation of the radiologist with 10 years of diagnostic experience. The radiomics features were extracted using Anaconda (version 4.2.0) with the PyRadiomics package (github.com/Radiomics/pyradiomics) according to the feature guidelines of the Image Biomarker Standardization Initiative (IBSI) (28).

### 2.6 Optimal radiomics signature construction

To improve the diagnostic performance and select the best radiomics model, an optimal radiomics model was constructed in the following two steps: i single-phase radiomics models: arterial phase or portal venous phase; ii combined-phase radiomics models: arterial phase and portal venous phase.

We employed the same strategy of feature selection and model construction above. The datasets were randomly divided into a training cohort and a test cohort with a case number ratio of 7:3. The patients were randomly divided into a training cohort (n = 89) and a test cohort (n = 37). The DeLong test was used to compare the differences of diagnostic performance among three radiomics models.

### 2.7 Clinical and clinical-radiomics diagnostic model construction

Twenty-one clinical factors and radiographic scores partially referenced from Kim et al. (29) and Cuiping Zhou et al. (30) including sex, age, max diameter, tumor location, growth pattern, ulceration, air density within the mass, surrounding fat space, tumor margin, tumor shape, direct organ invasion, density, calcification, intratumoral hemorrhage, necrosis, enlarged vessels around the tumor, apparent vessels in the tumor (AVT), enhancement pattern, lymphadenopathy, liver metastasis and level of enhancement were evaluated retrospectively by two radiologists independently and blinded to the type of GIST, with disagreements judged by a more senior radiologist. Detailed definitions of the above variables are provided in **Supplementary Text S3**. Then, clinical and radiographic scores were compared between patients with KIT exon 11 deletions and patients without KIT exon 11 deletions in the training and test cohorts and used to build a clinical model.

The clinical-radiomics model (combined model) was constructed by clinical model and radiomics model.

### 2.8 Model effectiveness evaluation

The area under the receiver operating characteristic (ROC) curve (AUC) was used to evaluate the diagnostic performance of the models constructed by the training cohort and test cohort, whereby the radiomics score (Radscore) was calculated via the formula built in the training cohort. The accuracy of the radiomics model was evaluated in both the training and test cohorts. The DeLong test was used to compare the diagnostic performance in the clinical model, radiomics model and combined model. The model calibration was assessed using calibration curves and the Hosmer–Lemeshow test. Decision curve analysis (DCA) was performed to estimate the clinical benefits of the models.

### 2.9 External validation

To ensure high replication of the model, diagnostic performances were evaluated of the radiomics model, clinical model, and combined model in the external cohort.

### 2.10 Survival risk stratification of GIST patients

The end point of follow-up is the disease-free survival (DFS) and overall survival (OS). DFS time starts from the date of surgery until the date of recurrence is determined. OS refers to the time from the date of surgery to the patient ‘s death or the last follow-up. Survival and recurrence information of patients were obtained through regular follow-up. Kaplan-Meier survival curves were used to assess disease-free survival (DFS) and overall survival (OS), and differences in survival time between groups were compared using the Log rank test. Risk score was calculated for each GIST patient by Radscore_AP-PVP_ so that to place patients into high or low risk subgroups.

### 2.11 Statistical analysis

Statistical analysis was mainly performed using R 3.6.1 (www.Rproject.org). Statistical analysis was performed using R 3.6.1 (www.Rproject.org). All the codes used in this research are available on the public website (https://github.com/martin18382076157/Levin-ma). The packages in R used in this study were tidyverse, caret,DMWR, mRMRe, glmnet, pROC, rmda, ggpubr, ModelGood, rms, and DescTOOLs.

The statistical approach in this study mainly involves the construction of radiomics label (Radscore), clinical model (Clinics), combined model (Combine) using Radscore and Clinics, model diagnostic performance evaluation, and external validation. Firstly, patients from our hospital are randomly divided into training and testing groups in a 7:3 ratio based on stratified randomization. Patients from other hospital are used as external validation data. The training group patients are used for radiomics feature dimension reduction, mRMR was used to reduction features redundancy to reduce overfitting. The least absolute shrinkage and selection operator (LASSO) algorithm is used with the minimum penalty coefficient to select the corresponding features for Radscore construction and 10-fold cross-validation was used to external validate the radiomics score.

During the clinical model building, the collinearity among clinical parameters is assessed using the variance inflation factor (VIF), and parameters with VIF< 5 are retained. The differences in demographic and clinical variables between patients with KIT exon 11 deletions and patients without KIT exon 11 deletions are evaluated using chi-square tests or Fisher’s exact tests for categorical data and t-tests (assuming equal variance and normal distribution) or rank-sum tests (if assumptions are not met) for continuous data. Parameters showing statistically significant differences are used to construct the clinical model using multiple logistic regression based the minimum Akaike information criterion (AIC) principle. The combined model, based on the selected clinical variables and the Radscore.

After model construction, model evaluation is performed using ROC analysis, Hosmer-Lemeshow test, and decision curve analysis (DCA).

Among the packages in R, the tidyverse and caret packages were used for data preprocessing and patient grouping, the DMWR package was used for SMOTE data handling, the mRMRe package was used for feature dimension reduction using mRMR analysis, the glmnet package was used for LASSO analysis in Radscore construction, the pROC package was used for ROC analysis, the rmda package was used for DCA analysis, the ggpubr package was used for data visualization, the rms package was used for nomogram plotting, the ModelGood package was used for model diagnostic performance evaluation.

## 3 Results

### 3.1 Characteristics and clinical features of the patients

Among the 126 GIST patients, 66 patients with KIT exon 11 deletions and 60 patients without deletions. The demographic and clinical variables of the patients in the training and test cohorts are summarized in **Table 2**. All the demographic data, including sex, age, and various clinical features, did not show significant differences between the training cohort and test cohort (P > 0.05) (**Table 3**).

**Table 2:** Demographic and clinical characteristics of patients in the training and test cohort.

	Training cohort (n=89)			Test cohort (n=37)		
	Without exon 11 deletion (n=42)	With exon 11 deletion (n=47)	P value	Without exon 11 deletion (n=18)	With exon 11 deletion (n=19)	P value
Sex			0.525			0.879
Female	18	16		9	8	
Male	24	31		9	11	
Age (y)	51.1 ± 11.0	54.0 ± 11.0	0.208	54.1 ± 12.4	59.2 ± 8.4	0.135
Max diameter (cm)	6.2 ± 3.0	8.7 ± 5.4	0.008	5.0 ± 2.9	8.3 ± 4.2	0.006
Location			0.059			–
Esophagus	0	2		0	0	
Stomach	22	24		8	9	
Duodenum	9	3		1	1	
Small intestine	8	17		8	9	
Colorectal	3	1		1	0	
Growth pattern			0.872			0.019
Endoluminal	8	7		4	1	
Exophytic	28	33		14	12	
Mixed	6	7		0	6	
Ulceration (no/yes)	33/9	38/9	0.998	16/2	13/6	0.266
Air density within the mass (no/yes)	32/10	34/13	0.864	14/4	9/10	0.117
Surrounding fat space (clear/unclear)	21/21	29/18	0.370	10/8	15/4	0.243
Tumor margin (well-defined/ill-defined)	24/18	14/33	0.017	9/9	6/13	0.420
Tumor shape (circular/irregular)	17/25	21/26	0.853	6/12	5/14	0.915
Direct organ invasion (no/yes)	40/2	45/2	1.000	18/0	19/0	–
Density (homogenous/heterogeneous)	10/32	14/33	0.693	6/12	5/14	0.915
Calcification (no/yes)	36/6	36/11	0.411	13/5	12/7	0.812
Intratumoral hemorrhage (no/yes)	39/3	43/4	1.000	18/0	17/2	0.491
Necrosis (no/yes)	15/27	10/37	0.202	8/10	3/16	0.148
Enlarged vessels around tumor (no/yes)	23/19	18/29	0.179	9/9	8/11	0.879
Apparent vessels in tumor (no/yes)	8/34	20/27	0.031	1/17	8/11	0.027
Enhancement pattern (no/yes)	16/26	15/32	0.698	5/13	4/15	0.926
Lymphadenopathy (no/yes)	39/3	38/9	0.179	15/3	14/5	0.754
Liver metastasis (no/yes)	38/4	43/4	1.000	15/3	15/4	1.000
Level of enhancement (mild/marked)	30/12	43/4	0.029	13/5	15/4	0.926

**Table 3:** Demographic and clinical characteristics of patients between the training and test cohort.

	Training (n=89)	Test (n=37)	p value
Sex			0.544
Female	34	17	
Male	55	20	
Age (y)	52.7 ± 11.1	56.7 ± 10.7	0.058
Max diameter (cm)	7.5 ± 4.6	6.7 ± 4	0.359
Location			0.263
Esophagus	2	0	
Stomach	46	17	
Duodenum	12	2	
Small intestine	25	17	
Colorectal	4	1	
Growth pattern			0.887
Endoluminal	15	5	
Exophytic	61	26	
Mixed	13	6	
Ulceration (no/yes)	71/18	29/8	1.000
Air density within the mass (no/yes)	66/23	23/14	0.258
Surrounding fat space (clear/unclear)	50/39	25/12	0.324
Tumor margin (well-defined/ill-defined)	38/51	15/22	0.980
Tumor shape (circular/irregular)	38/51	11/26	0.246
Direct organ invasion (no/yes)	85/4	37/0	0.452
Density (homogenous/heterogeneous)	24/65	11/26	0.923
Calcification (no/yes)	72/17	25/12	0.166
Intratumoral hemorrhage (no/yes)	82/7	35/2	0.914
Necrosis (no/yes)	25/64	11/26	0.954
Enlarged vessels around tumor (no/yes)	41/48	17/20	1.000
Apparent vessels in tumor (no/yes)	28/61	9/28	0.558
Enhancement pattern (no/yes)	31/58	9/28	0.345
Lymphadenopathy (no/yes)	77/12	29/8	0.384
Liver metastasis (no/yes)	81/8	30/7	0.206
Level of enhancement (mild/marked)	73/16	28/9	0.570

### 3.2 Feature selection and signature construction

A total of 1218 radiomics features were extracted from both the arterial and portal venous phase images. The intraobserver and interobserver ICCs all indicated favorable feature extraction reproducibility (mean ICC > 0.85). A total of 247 radiomics features with consistency values less than 0.7 were removed, leaving 971 features. More details on the deleted and selected features are presented in the **Supplementary Materials** (Excel. Feature delete and feature select).

**Figure 2:**
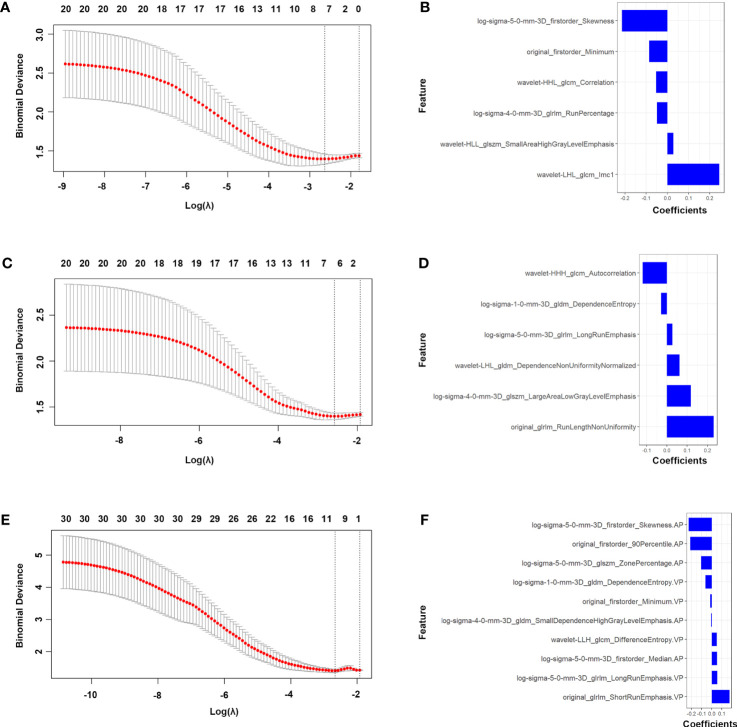
Features selection of the AP, PVP and AP-PVP radiomics model. LASSO [**(A)** in AP radiomics model, **(C)** in PVP radiomics model and **(E)** in AP-PVP radiomics model]. Coefficients of features in RadscoreAP **(B)**, RadscorePVP **(D)** and RadscoreAP-PVP **(F)**. AP, arterial phase; PVP, portal venous phase.

Based on the principles of least penalty coefficient and least binomial deviation, Radscore_AP_ was constructed with 6 features corresponding to minimum logλ=0.072 in the arterial phase (**Figures 2A, B**). Radscore_PVP_ was constructed with 6 features corresponding to minimum logλ=0.076 in the portal venous phase (**Figures 2C, D**). Radscore_AP-PVP_ was constructed with 10 features in both phases corresponding to minimum logλ=0.069 (**Figures 2E, F**). The formula is as follows:


$$\begin{array}{l} {\text{Radscore}}_{\text{AP}-\text{PVP}}\\ \;=-0.211\times \text{original}\_\text{firstorder}\_90\text{Percentile}.\text{AP}-0.103\\ \;\times \text{log}\_\text{sigma}\_5\_0\_\text{mm}\_3\text{D}\_\text{glszm}\_\text{ZonePercentage}.\text{AP}-0.061\\ \;\times \text{log}\_\text{sigma}\_1\_0\_\text{mm}\_3\text{D}\_\text{gldm}\_\text{DependenceEntropy}.\text{PVP}-0.228\\ \;\times \text{log}\_\text{sigma}\_5\_0\_\text{mm}\_3\text{D}\_\text{firstorder}\_\text{Skewness}.\text{AP}+0.05\\ \;\times \text{wavelet}\_\text{LLH}\_\text{glcm}\_\text{DifferenceEntropy}.\text{PVP}-0.005\\ \;\times \text{log}\_\text{sigma}\_4\_0\_\text{mm}\_3\text{D}\_\text{gldm}\_\text{SmallDependenceHighGrayLevelEmphasis}.\text{AP}\\ \;+0.054\times \text{log}\_\text{sigma}\_5\_0\_\text{mm}\_3\text{D}\_\text{glrlm}\_\text{LongRunEmphasis}.\text{PVP}+0.053\\ \;\times \text{log}\_\text{sigma}\_5\_0\_\text{mm}\_3\text{D}\_\text{firstorder}\_\text{Median}.\text{AP}+0.175\\ \;\times \text{original}\_\text{glrlm}\_\text{ShortRunEmphasis}.\text{PVP}-0.015\\ \;\times \text{original}\_\text{firstorder}\_\text{Minimum}.\text{PVP}+0.12 \end{array}$$

### 3.3 Optimized Radscore construction

**Figure 3:**
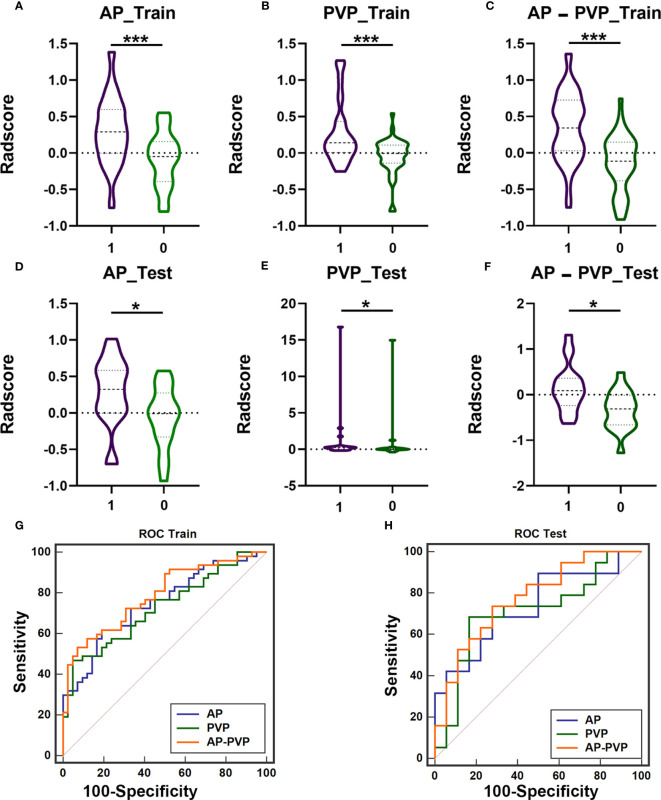
The difference of Radscores between training cohort and test cohort, and diagnostic efficacy of the radiomics models in the training cohort and test cohort. The radiomics score of arterial phases **(A, D)**, portal venous phase **(B, E)**, arterial-portal venous phase **(C, F)** showed significant differences between patients with KIT exon 11 deletion (1, purple) and without KIT exon 11 deletion (0, green) in the training cohort and test cohort (*** P<0.001, * P<0.05). ROC curve of radiomics model in the training cohort (**G**, AP-PVP model performed best: AUC = 0.787) and test cohort (**H**, AP-PVP model performed best: AUC = 0.775). ROC, receiver operating characteristic; AUC, area under the curve; AP, arterial phase; PVP, portal venous phase.

First, we investigated and compared the arterial phase, portal venous phase, and arterial-portal venous phase separately. All the radiomics score showed significant differences between patients with mutation and without mutation (**Figures 3A–F**) in the training cohort (P_AP_<0.001, P_PVP_<0.001, and P_AP-PVP_<0.001, respectively) and test cohort (P_AP_=0.014, P_PVP_=0.017, and P_AP-PVP_=0.004, respectively). The AUCs of the Radscore model in the training cohort and in the test cohort were greater than 0.7 (ROC and diagnostic performance of AP, PVP and AP-PVP are shown in **Figures 3G, H** and **Table 4**). The arterial-portal venous phase was the optimized model with the highest diagnostic performance in the training cohort and test cohort (AUC = 0.787 vs. 0.775). However, there were no statistically significant differences among the radiomics models in either the training cohort (M_AP_ vs. M_PVP_, P=0.775; M_AP_ vs. M_AP-PVP_, P=0.563; M_PVP_ vs. M_AP-PVP_, P=0.388) or the test cohort (M_AP_ vs. M_PVP_, P=0.867; M_AP_ vs. M_AP-PVP_, P=0.725; M_PVP_ vs. M_AP-PVP_, P=0.596).

**Table 4:** Diagnostic performance of radiomics in the arterial phase, portal venous phase, and arterial-portal venous phase separately.

	AP		PVP		AP-PVP	
	Training cohort	Test cohort	Training cohort	Test cohort	Training cohort	Test cohort
AUC	0.747	0.734	0.725	0.711	0.787	0.775
95% CI						
Lower	0.643	0.563	0.621	0.538	0.687	0.608
Upper	0.833	0.865	0.815	0.847	0.866	0.895
Accuracy	0.707	0.649	0.696	0.703	0.719	0.623
95% CI						
Lower	0.609	0.475	0.590	0.530	0.614	0.447
Upper	0.799	0.748	0.790	0.841	0.809	0.775
Sensitivity	0.617	0.579	0.468	0.578	0.532	0.316
95% CI						
Lower	0.464	0.335	0.302	0.335	0.381	0.126
Upper	0.755	0.797	0.599	0.797	0.679	0.566
Specificity	0.810	0.722	0.952	0.833	0.929	0.944
95% CI						
Lower	0.464	0.335	0.838	0.586	0.805	0.727
Upper	0.755	0.797	0.994	0.964	0.985	0.999
PPV	0.784	0.687	0.917	0.786	0.893	0.857
NPV	0.654	0.619	0.615	0.652	0.639	0.566

AP, arterial phase; PVP, portal venous phase; AUC, area under the curve; CI, confidence interval; PPV, positive predicted value; NPV, negative predicted value.

### 3.4 Clinical model and combined model of the arterial-portal venous phase

Maximum diameter of the tumor, tumor margin, and AVT were identified as independent factors for the clinical prediction model by VIF< 5 based on clinical variables and the minimum AIC principle. The VIFs for the three clinical characteristics were 3.5, 2.7 and 4.3, and the clinical model was developed based on these factors.

**Figure 4:**
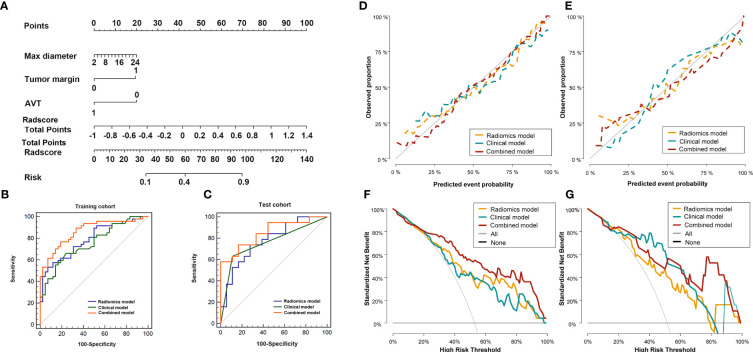
Diagnostic evaluation, calibration curves and decision curve analysis of the radio-genomics, clinical and combined models of AP-PVP. Nomogram based on the combined model **(A)**. ROC curves of the radio-genomics, clinical and combined models in the training cohort **(B)** and test cohort **(C)**; The combined model performed best (AUC of the training cohort and test cohort, 0.863 vs.0.851). Calibration curves in the training cohort (**D**, P=0.258) and test cohort (**E**, P=0.084). DCA of in the training cohort **(F)** and test cohort **(G)**. Combined model (red line) performs best. The X-axis indicates the threshold probability. The Y-axis shows the model benefit. The red line represents the combined model. The orange line represents the radiomics score (Radscore), and the blue line represents the clinical model.


The combined model included three clinical parameters (maximum diameter of the tumor, tumor margin, and AVT) and Radscore_AP-PVP_, the nomogram of the combined model is shown in **Figure 4A**, and the formula is as follows:

Nomogram score=−0.402+0.057×max diameter+1.348×tumor margin−1.380×AVT+2.881×Radscore

The cut-off of the formula is 0.217.

### 3.5 Diagnostic performance of the radiomics model, clinical model and combined model of the arterial -portal venous phase

The discriminatory efficiency of the radiomics model, clinical model and combined model was assessed using ROC analyses (**Figures 4B, C**, **Table 5**). In the training cohort, the diagnostic performance of the combined model was significantly higher than that of the radiomics model (P=0.026) and the clinical model (P=0.006). In the test cohort, the combined model showed the highest diagnostic performance (AUC_combined_ = 0.851, 95% CI = 0.695-0.946), but there was no significant difference in comparison with the radiomics model (P = 0.183) and the clinical model (P = 0.151).

**Table 5:** Diagnostic performance of radio-genomics, clinical and combined models.

	Radiomics model		Clinical model		Combined model	
	Training cohort	Test cohort	Training cohort	Test cohort	Training cohort	Test cohort
AUC	0.787	0.775	0.753	0.760	0.863	0.851
95% CI						
Lower	0.687	0.608	0.651	0.592	0.774	0.695
Upper	0.866	0.895	0.839	0.885	0.927	0.946
Accuracy	0.719	0.622	0.708	0.757	0.787	0.730
95% CI						
Lower	0.614	0.448	0.602	0.588	0.687	0.559
Upper	0.809	0.775	0.799	0.882	0.866	0.862
Sensitivity	0.532	0.316	0.660	0.632	0.766	0.846
95% CI						
Lower	0.381	0.126	0.507	0.384	0.620	0.604
Upper	0.679	0.566	0.791	0.837	0.877	0.966
Specificity	0.929	0.944	0.762	0.889	0.810	0.667
95% CI						
Lower	0.805	0.727	0.605	0.653	0.659	0.410
Upper	0.985	0.999	0.879	0.986	0.914	0.867
PPV	0.893	0.857	0.756	0.857	0.818	0.756
NPV	0.639	0.567	0.667	0.696	0.579	0.889

All mutations identified and the results of the models are provided in **Supplementary Table S1**.

### 3.6 Evaluation of the radiomics model, clinical model, and combined model

The Hosmer–Lemeshow test for the radiomics model, clinical model, and combined model showed that the combined model fit the data well, with no significant difference between the training cohort (P = 0.258) and the test cohort (P = 0.084) (**Figures 4D, E**).

The DCAs for the radiomics model, clinical model, and combined model in the training and test cohorts are shown in **Figures 4F, G**. DCA indicated the threshold probability of patients who under 100% will maximize the benefit. The net benefit for each model at various threshold probabilities are provided in **Supplementary Table S2**.

### 3.7 External validation of the combined model

**Figure 5:**
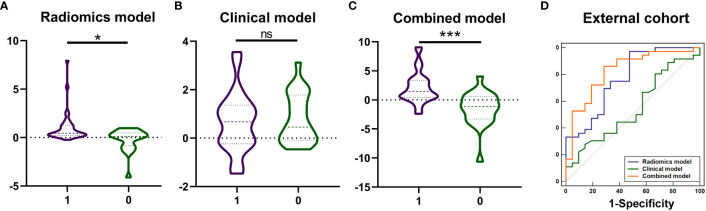
The values of model prediction and diagnostic performance of the radiomics, clinical and combined models in the external cohort. The values of model prediction of radiomics model **(A)**, and combined model **(C)** showed significant differences (*** P <0.001, * P <0.05, ns means no significance) between patients with KIT exon 11 deletion (1, purple) and without KIT exon 11 deletion (0, green). The values of model prediction of clinical model (**B**, P=0.546) were higher in patients with KIT exon 11 deletion than patients without KIT exon 11 deletion but didn’t show significant differences. The ROC curve of combined model **(D)** yielded the largest AUC of 0.828.

The values of radiomics model (**Figure 5A**), and combined model (**Figure 5C**) showed significant differences (P <0.05) between patients with KIT exon 11 deletion and without KIT exon 11 deletion in external valisation. The values of clinical model (**Figure 5B**, P=0.546) were higher in patients with KIT exon 11 deletion than patients without KIT exon 11 deletion but didn’t show significant differences. The combined model (**Figure 5D**, **Table 6**) yielded the largest AUC of 0.828 (95% CI, 0.705–0.915). Demographic and clinical characteristics of patients in the external cohort are provided in **Table 7**.

**Table 6:** Diagnostic performance of the radio-genomics, clinical and combined models in the external cohort.

	Radiomics model	Clinical model	Combined model
AUC	0.772	0.548	0.828
95% CI			
Lower	0.642	0.411	0.705
Upper	0.873	0.681	0.915
Accuracy	0.807	0.456	0.807
95% CI			
Lower	0.681	0.324	0.681
Upper	0.900	0.593	0.900
Sensitivity	0.972	0.806	0.861
95% CI			
Lower	0.855	0.640	0.705
Upper	0.999	0.918	0.953
Specificity	0.524	0.333	0.714
95% CI			
Lower	0.298	0.146	0.478
Upper	0.743	0.570	0.887
PPV	0.761	0.667	0.838
NPV	0.909	0.467	0.750

AUC, area under the curve; CI, confidence interval; PPV, positive predicted value; NPV, negative predicted value.

**Table 7:** Demographic and clinical characteristics of patients in the external cohort.

	External cohort (n=57)		
	Without exon 11 deletion(n=21)	With exon 11 deletion(n=36)	P value
Gender			0.654
Female	9	19	
Male	12	17	
Age (y)	55.1 ± 11.9	55.1 ± 9.8	0.997
Max-diameter (cm)	7.1 ± 4.0	7.9 ± 4.1	0.493
Location			0.007
Esophagus	0	0	
Stomach	16	19	
Duodenum	3	1	
Small intestine	0	14	
Colorectal	2	2	
Growth pattern			0.865
Endoluminal	6	8	
Exophytic	9	17	
Mixed	6	11	
Ulceration (No/Yes)	16/5	28/8	1.000
Air density within the mass (No/Yes)	16/5	29/7	0.958
Surrounding fat space (clear/unclear)	18/3	26/10	0.399
Tumor margin(well-defined/ill-defined)	8/13	17/19	0.694
Tumor shape(circular/irregular)	6/15	11/25	1.000
Direct organ invasion(no/yes)	18/3	28/8	0.701
Density(homogenous/heterogeneous)	11/10	19/17	1.000
Calcification(no/yes)	18/3	35/1	0.270
Intra-tumoral hemorrhage(no/yes)	21/0	36/0	0.047
Necrosis(no/yes)	10/11	16/20	1.000
Enlarged vessels around tumor(no/yes)	18/3	29/7	0.894
Apparent vessels in tumor(no/yes)	16/5	22/14	0.382
Enhancement pattern(no/yes)	6/15	10/26	1.000
Lymphadenopathy(no/yes)	19/2	32/4	1.000
Liver metastasis(no/yes)	21/0	36/0	0.047
Level of enhancement(mild/marked)	12/9	20/16	1.000

### 3.8 Prognostic stratification of GIST patients

**Figure 6:**
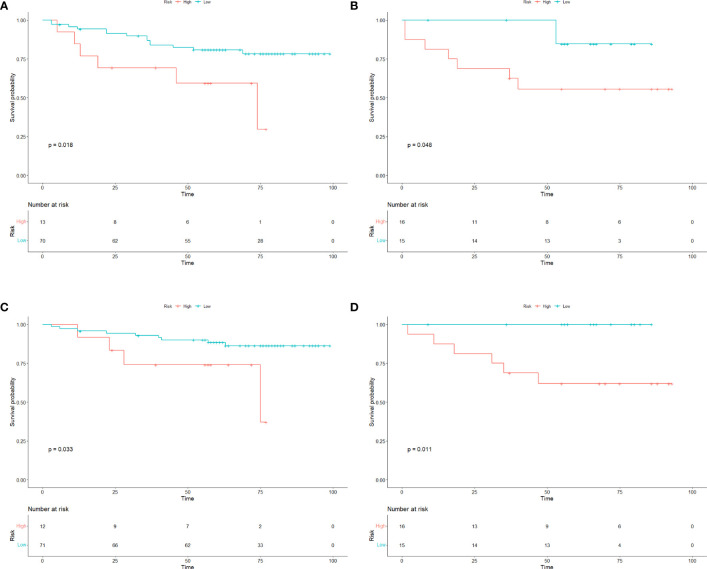
Survival curves of GIST patients. The Kaplan–Meier curves for DFS and OS in GIST patients in the **(A, C)** training cohorts and **(B, D)** test cohorts according to the different risk groups of Radscore. There was a significant difference in DFS and OS between the high-risk and low-risk groups in both the training and test cohorts (P<0.05). OS overall survival; DFS disease−free survival; GIST gastrointestinal stromal tumors.

As of Jun. 2023, 91.3% (115/126) GIST patients had completed the PFS and OS follow-up. Among them, 29 GIST patients experienced tumor recurrence during the follow-up, and 19 GIST patients died. Patients were divided into low-risk and high-risk subgroups in the training and test cohorts. In training cohort, the median DFS time was 46 months (high risk subgroup) and 62 months (low risk subgroup), the median OS time was 57 months (high risk subgroup) and 70 months (low risk subgroup); In test cohort, the median DFS time was 48 months (high risk subgroup) and 65 months (low risk subgroup), the median OS time was 62 months (high risk subgroup) and 66 months (low risk subgroup). Kaplan-Meier analysis showed that the DFS and OS curves of the low- and high-risk subgroups were significantly different in both the training and test cohorts (log rank test, p< 0.05), respectively (**Figure 6**).

### 3.9 Case presentation of model application

**Figure 7:**
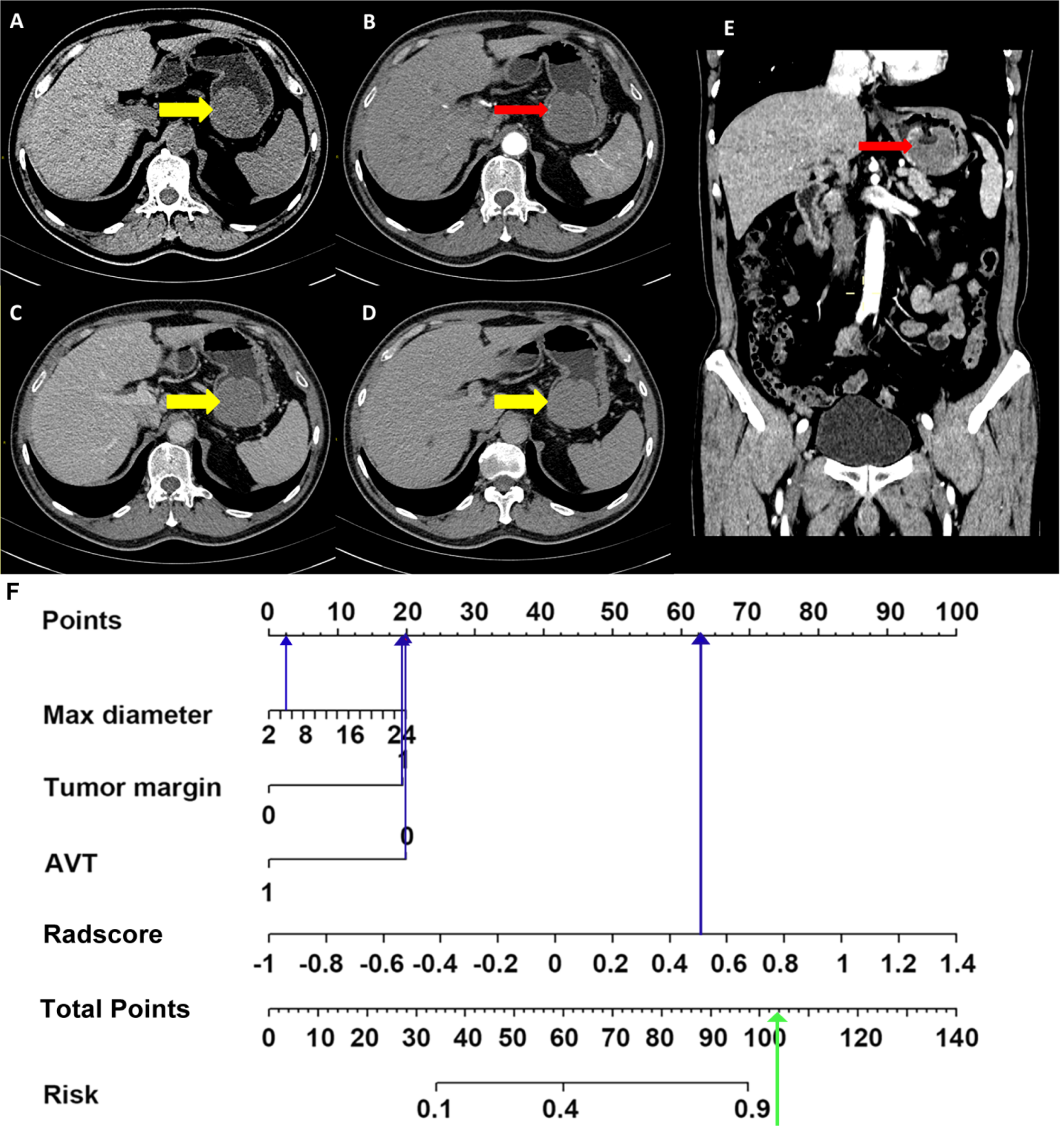
Application of combined model. Non-enhanced CT image **(A)**, arterial phase images (**B**, transverse section; **E**, coronal section), portal venous phase image **(C)**, delayed phase image **(D)**, nomogram **(F)**. A 58-year-old male was seen 1 week after the discovery of blood in the stool. Enhanced abdominal CT image shows a soft tissue density mass in the stomach (**A**, thick yellow arrows in CT images), with 5 cm diameter and unsmooth margin, no obvious enhanced vascular was seen in tumor (**B, E**, thin red arrows). The max diameter, tumor margin, AVT and Radscore points were substituted into the nomogram (blue arrow in **Figure 7F**) to obtain the total points. It was found that the probability of predicting KIT exon 11 deletion was greater than 90% (green arrow in **Figure 7F**). The pathological results of the patient showed KIT exon 11 deletion.

In our combined model, three clinical variables and the Radscore (AP-PVP) were used to predict the presence of preoperative KIT exon 11 deletion. As an example, a 58-year-old male patient was seen 1 week after the discovery of blood in the stool (**Figure 7**). The maximum diameter of the lesion was 5 cm, the tumor margin was assigned as 1 (ill-defined), the AVT was assigned as 0, and the Radscore was 0.553. When the above information was taken into the combined model formula, the score was 2.827, which was greater than the cutoff value of 0.217, the nomogram showed that the probability of KIT exon 11 deletion was greater than 90%. The pathological results of the patient showed KIT exon 11 deletion.

## 4 Discussion

In this work, we showed radiomics method based on enhanced CT images could predict KIT exon 11 mutation in patients with GIST, and the radiomics integrate AP and PVP images could identify KIT exon 11 deletion GIST with high sensitivity. Furthermore, we developed and validated a combined model that incorporated Radscore and maximum diameter of the tumor, tumor margin, AVT, which exhibited high accuracy for preoperatively predicting KIT exon 11 mutation. Importantly, the conglomerate of the radiomics and clinical risk factors in our combined model provides a straightforward, noninvasive, and mighty approach for personalized prediction of KIT exon 11 mutation before surgery. Precision medical research has successfully used radiomics methods to evaluate image attributes, and to predict tumor genotypes (31–33). This represents the study focused on the clinical-radiomics analysis on enhanced CT imaging for preoperative prediction of KIT exon 11 deletion of GISTs.

Recently, the postoperative indicators including location, size, morphology, immunohistochemistry, and molecular genetics have been examined for the malignant potential of GISTs (34). GIST with KIT exon 11 deletions exhibit higher proliferation rates and shorter disease-free survival times compared with GISTs with other KIT exon 11 mutations (35). Previous study (36) evaluating 1303 patients with GISTs showed that tumor size >5 cm was significantly correlated with the increased rate of tumor recurrence. Tumor size had also been found to be of important diagnostic value in the risk classification of GISTs, irrespective of the NIH standard, AFIP standard, or AJCC staging system. In our study, compared to GISTs without KIT exon 11 deletion, GISTs with KIT exon 11 deletion usually exhibit characteristics such as a larger tumor maximum diameter, an ill-defined tumor margin. Prominent tumor vasculature, and more obvious tumor vessels more likely to occur in the GIST with KIT exon 11 deletions, this is consistent with previous research and suggesting a relationship between the deletion of KIT exon 11 and biological behavior of aggression (37). GISTs with different types of mutations exhibit different therapeutic effects, and prognosis (38). exon 11 mutant GISTs are usually sensitive to imatinib, imatinib therapy for 3 years after surgery significantly improved the prognosis of patients with GISTs with KIT exon 11 deletion (18). Therefore, deletion of KIT exon 11 should be a required assessment to explore a more appropriate treatment strategy for GIST patients.

The arterial-portal venous phase radiomics signature had the greatest performance, and previous research generated equivalent findings (39). The performance of diagnostics of the clinical model was lower to that of the Radscore model. The combined model had better predictive effectiveness and clinical applicability with the validation set than the radiomics nomograms, which indicated that the judgment efficiency of the combined feature analysis was superior to that of the Radscore texture analysis or the clinical feature analysis alone. The radiomics reflected molecular-level pathology better than the clinical factors, which confirmed the enormous potential of the radiomics to distinguish GISTs with the KIT exon 11 deletions.

In the past, radiomics was able to convert images into high-throughput quantitative data that may identify intratumor heterogeneity and correlate with gene expression levels. On enhanced CT images, Xu et al. (40) presented the evidence that CT texture analysis may help distinguish GISTs with KIT exon 11 mutation from those without KIT exon 11 mutation. They discovered that the standard deviation of the textural parameters of tumors lacking the KIT exon 11 mutation is an independent predictor of the absence of the mutation. Liu et al. (41) evaluated the ability of three alternative models (model_[CT]_, model_[radiomics + clinical]_, and model_[CT + radiomics + clinical]_) to distinguish between GISTs and those without KIT exon 11 mutation. In our work, we focused on the performance of diagnostics of GISTs with and without KIT exon 11 deletion. 1218 features for radio-genomics, followed by more typical CT image features such as morphological and density variables. Patients with GIST who had previously received anticancer treatment (such as TKI treatment or surgery) were excluded from the research. The model’s trustworthiness is enhanced by the incorporation of an external validation cohort (with an AUC of 0.829).

Radiomics model could effectively stratify the risk level of GIST patients, which is consistent with previous studies (42, 43), thus allowing better preoperative prediction of patient recurrence or mortality.

Nevertheless, the current research has a few limitations. The retrospective approach and patient exclusion criteria may introduce a certain selection bias. Due to the small size of sample, we merged GISTs with gene mutations except for without KIT exon 11 deletion. It is vital to distinguish between distinct mutation types. Future research with a large size of sample or a specific design should take this into consideration. Currently, Radscore does have the problem of interpreting the correlation between radiomics features and physiological characteristics of diseases, how to correlate the both of them will be an important research for radiomics, and it is also the subsequent research of this research. Some studies illustrated the correlation of some radiomics features with diseases, such as entropy and energy (44–47), so in the future we will focus on exploring the relevance of more features to disease pathology. And finally, four distinct scanners were used for the CT scans. Nevertheless, the diversity of machines can make the model more repeatable, thereby promoting their widespread use.

## 5 Conclusions

In conclusion, clinical-radiomics based on enhanced CT imaging provides a good predictive capacity for GISTs with and without KIT exon 11 deletion. Given that CT imaging is frequently used in all phases of GIST diagnosis and treatment and provides a noninvasive opportunity to detect gene mutation types, this method may have a substantial impact.

## Data availability statement

The raw data supporting the conclusions of this article will be made available by the authors, without undue reservation.

## Ethics statement

The studies involving humans were approved by the Ethics Committee of Tongji Medical College of Huazhong University of Science and Technology. The studies were conducted in accordance with the local legislation and institutional requirements. The participants provided their written informed consent to participate in this study.

## Author contributions

Guarantors of integrity of entire study, PH, YZ, QL. Study concepts/study design or data acquisition or data analysis/interpretation, YZ, XY, PZ, YYZ, KT. Manuscript drafting or manuscript revision for important intellectual content, all authors. Manuscript final version approval, all authors. Literature research, ND, GM, YL. Statistical analysis, LW, LM. Manuscript editing, XY, PH, QL. All authors contributed to the article and approved the submitted version.
