# Supplementary material for: Clinical-radiomics-based treatment decision support for KIT Exon 11 deletion in gastrointestinal stromal tumors: a multi-institutional retrospective study
Source: Front Oncol. 2023 Aug 14;13:1193010. doi: 10.3389/fonc.2023.1193010 (PMC10461453; doi:10.3389/fonc.2023.1193010)
Supplement: Supplementary file 1 [file DataSheet_1.zip › text S1-3.docx]

**Text S1 Genetic Testing of patients**

Sequence of Gene was extracted from formalin-fixed paraffin-embedded or fresh frozen tumor tissue. Then after DNA extraction, sequence was analyzed by Sanger sequencing. We have analyzed exons 9, 11, 13 and 17 of the KIT gene. Deletions and substitutions or duplications/insertions, the amino acid of sequence have changed not only order but also number, were defined as mutation in this study.

An indel refers to a deletion of amino acids and a concomitant insertion of at least one amino acid, whereas a deletion is an exclusive deletion of at least one amino acid.

## Text S2 Image Preprocessing and Image Quantization

The intensity of the signal of CT haven’t been considered as the absolute value of patients, images normalization should be done to reduce the bias of imaging parameters. The equation of image standardization is as follows:

$$image standardization=\frac{x-\mu}{adjusted <uscore>stddv}$$

$${adjusted}_{stddev}=max\left( \sigma,\frac{1.0}{\sqrt{N}} \right)$$

μ indicates the average value of the signal in CT, x means the matrix, σ indicates the standard deviation of intensity of images, N means the detail number of voxels and pixel in images not only ROI. The normalized equation of images is as follows:

$$normalize=\frac{x_{i}-min\left( x \right)}{max\left( x \right)-min\left( x \right)}$$

Xi means the value of CT signal of some pixel or voxel. Min(x) indicates the minimum value of CT signal for all voxels. Max(x) indicates the maximum value of CT signal for all voxels.

During the quantization of images, all gray values of voxel in CT images should converse into a discrete group of gray values[1]. According to the example setting of PyRadiomics (https://github.com/AIM- Harvard/pyradiomics/tree/master/examples/example Settings), we fixed a bin width with 5 to quantized CT images. According to the previous study, different images modality should be pre-processed with proper bin width to keep the bins between 16 and 128. In the PyRadiomics, we can calculate a parameter named by first order range to find the suitable bin width. An absolute discretization was calculated with a fixed bin size (binsize = 5) such that the new bin had been assigned to voxel intensities to each BS gray level starting from 0. The equation is as follows:

$$I_{BS}\left( X \right)=\left[ \frac{I\left( x \right)}{BS} \right]-min\left( \left[ \frac{I\left( x \right)}{BS} \right] \right)+1$$

I(x) mean the intensity of the voxel in the ROI; BS means binsize, and IBS(x) presented the gray value of some voxel that had been discretized [2].

After this, all original CT images have been transformed into Gaussian and Wavelet images. During the wavelet transformation, each level has 8 decompositions. The setting of 8 decompositions were as follows:

1. level [0]: integer, 0 based level of wavelet that level [0] is the first decompositions to calculate a signature. 2) Level [1]: integer, the firs wavelet decompositions to calculate a signature. 3)Wavelet [“coif1”]: string, means the type for wavelet decomposition which is enumerated value.

The transformation of gaussian images were calculated by convolving the image with the second derivative (Laplacian) of a Gaussian kernel. The Gaussian kernel could be calculated as follows:

$$G\left( x,y,z,\sigma\right)=\frac{1}{\left( \sigma\sqrt{2\pi} \right)^{3}}e^{-\frac{x^{2}+y^{2+z^{2}}}{2\sigma^{2}}}$$

The Gaussian kernel was convolved by the ∇2G[3].

**Text S3 definitions of the variables**

(a)The max diameter is defined as the maximum length measured on transverse or coronal CT images. (b) Tumor location was classified as esophagus, stomach, duodenum, small intestine and colorectal. (c) Growth pattern was categorized as endoluminal, exophytic and mixed on CT images. Entire tumor in the intestinal or stomach cavity is considered to be endoluminal; entire tumor outside the intestinal or gastric lumen is considered to be exophytic; tumor growth across the bowel or stomach wall is considered to be mixed. (d) Ulceration was classified as presence of ulcer crater or absence of ulcer crater [4]. (e) Air density within the mass was classified as the presence of air density in the tumor or absence of air density in the tumor. (f) Surrounding fat space was classified as clear of the fat space or unclear of the fat space. (g) Tumor margin was categorized as well-defined (round or oval tumors with a smooth contour) or ill-defined (confluent multinodular or nodular with extranodular growth). (h) Tumor shape was evaluated as circular or irregular. (i) Direct organ invasion was considered to be the direct invasion of the tumor into other organs. (j) Density was categorized as homogenous or heterogeneous. (k) Calcification was classified as the presence of calcification or absence of calcification in the tumor. (l) Intratumoral hemorrhage was defined as the presence of high-density hemorrhagic foci observed within the tumor. (m) Necrosis was classified as presence of calcification or absence of necrosis in the tumor. (n) Enlarged vessels around the tumor was defined as the presence of thickened blood vessels surrounding the tumor with persistent enhancement. (o) Apparent vessels in the tumor were defined as the persistence of discrete arterial enhancement within the tumor in the arterial phase. (p) Enhancement pattern was classified as homogenous or heterogeneous on enhanced imaging. (q) Lymphadenopathy was classified as the presence or absence of peripheral enlarged lymph nodes. (r) Liver metastasis was classified as the presence or absence of liver metastases. (s) Level of enhancement was defined as the degree of tumor enhancement observed in the arterial phase, classified as mild or marked enhancement.

References

[1] E.P.V. Le, L. Rundo, J.M. Tarkin, N.R. Evans, M.M. Chowdhury, P.A. Coughlin, H. Pavey, C. Wall, F. Zaccagna, F.A. Gallagher, Y. Huang, R. Sriranjan, A. Le, J.R. Weir-McCall, M. Roberts, F.J. Gilbert, E.A. Warburton, C.B. Schonlieb, E. Sala, and J.H.F. Rudd, Assessing robustness of carotid artery CT angiography radiomics in the identification of culprit lesions in cerebrovascular events. Sci. Rep. 11 (2021) 3499.

[2] L. Duron, D. Balvay, S. Vande Perre, A. Bouchouicha, J. Savatovsky, J.C. Sadik, I. Thomassin-Naggara, L. Fournier, and A. Lecler, Gray-level discretization impacts reproducible MRI radiomics texture features. PLoS One 14 (2019) e0213459.

[3] J.J.M. van Griethuysen, A. Fedorov, C. Parmar, A. Hosny, N. Aucoin, V. Narayan, R.G.H. Beets-Tan, J.C. Fillion-Robin, S. Pieper, and H. Aerts, Computational Radiomics System to Decode the Radiographic Phenotype. Cancer Res. 77 (2017) e104-e107.

[4] S. Bano, S.K. Puri, L. Upreti, V. Chaudhary, H.K. Sant, and R. Gondal, Gastrointestinal stromal tumors (GISTs): an imaging perspective. Japanese journal of radiology 30 (2012) 105-15.
